# Editorial: Mechanical Loading and Bone

**DOI:** 10.3389/fendo.2015.00184

**Published:** 2015-12-14

**Authors:** Jonathan H. Tobias

**Affiliations:** ^1^Musculoskeletal Research Unit, School of Clinical Sciences, University of Bristol, Bristol, UK

**Keywords:** mechanical loading, skeleton, accelerometry, osteoblast, osteoporosis

**The Editorial on the Research Topic**

**Mechanical Loading and Bone**

## Introduction

This Research Topic comprises 11 interesting and topical articles describing a mixture of clinical and laboratory approaches to how mechanical loading influences the skeleton. Of the five clinical papers, those by Tobias et al. and Janz et al. address important methodological issues concerning the use of accelerometers to record physical activity in a way that is relevant to mechanical loading of bone. Two separate papers by Toshihiro Sugiyama et al. discuss how understanding of skeletal responses to mechanical loading can help interpret the mechanism of action of bone anabolic drugs. The paper by Zengin et al. illustrates how understanding of mechanical loading responses can be used to interpret ethnic differences in skeletal structure. Of the six laboratory papers, those by Meakin et al. and Vazquez et al. focus on methodological approaches to study mechanical loading *in vivo* and *in vitro*, respectively. Four of the papers explore the mechanisms involved in mediating different aspects of mechanical loading responses of bone. García-López et al. report their findings concerning the mechanisms involved in the inhibition of bone resorption by cyclical mechanical strain *in vitro*; Kang and Robling review the role of the Wnt pathway in mechanotransduction; Alzahrani et al. review the mechanisms involved in distraction osteogenesis; and Betts and Müller review theories and models of mechanical regulation of bone regeneration after bone injury.

## Clinical Papers

The papers by Tobias and Janz represent contrasting approaches to study the relationships between physical activity and bone measures based on accelerometry. While the former addresses the role of physical activity in preserving bone in older people, and the latter examines its role in bone accrual in childhood. Moreover, these papers utilize distinct approaches to extract relevant information about mechanical loading from accelerometry data. Janz et al. use conventional counts per minute thresholds but apply a relatively high threshold denoting vigorous PA on the basis that such activity is more likely to be osteogenic, as borne out by their results. By contrast, Tobias et al. extracted the number of counts beyond specific *g* thresholds on the basis that these are more directly related to ground reaction forces.

The remaining three clinical papers illustrate how understanding of mechanical loading responses can be used to explain findings from epidemiological to interventional studies of the skeleton. In their hypothesis article on teriparatide effects on bone, Toshihiro Sugiyama et al. suggest that the reason why gains in bone at the outer surface are limited is because simultaneous gains at the inner surface act to limit strain at the outer surface. In a further hypothesis article, the same authors, Sugiyama et al. suggest that the anti-sclerostin inhibitors blosozumab and romosozumab are particularly effective at simulating bone formation as a consequence of their ability to overcome negative feedback of the mechanostat by interfering with this process due to its dependence on sclerostin. In their review of ethnic differences in bone health, Ayse Zengin et al. conclude that differences in fracture rate between ethnic groups cannot adequately be explained by differences in areal bone mineral density, suggesting the need to describe these based on more detailed measures of bone structure. Differences in the latter may in turn reflect ethnic differences in muscle mass and force, or alternatively how the skeleton responds to mechanical loading by these factors.

## Laboratory Papers

The papers by Meakin et al. and Vazquez et al. describe contrasting methodological approaches to studying the mechanisms that mediate mechanical loading responses. Meakin et al. describe how use of *in vivo* studies based on a range of animal models has contributed to our understanding of the type of strain stimulus to which the skeleton is most responsive. Vazquez focuses on *in vitro* models of mechanical strain responses, describing a novel 3D osteocyte–osteoblast co-culture system.

The remaining four laboratory papers describe different aspects of the mechanisms involved in mechanical loading responses. García-López et al. report that cyclical strain of osteoblast monolayers reduces expression of the bone resorptive cytokine RANKL, but surprisingly other cytokines that might be expected to mediate this response show a paradoxical increase in expression. The review by Kang and Robling focuses on the role of Wnt signaling in mediating osteogenic responses to mechanical strain and suggests that while LRP5 is known to be involved, the closely related receptor LRP6 may also play a hitherto unrecognized role.

In their review article, Alzahrani et al. discuss how characterization of the molecular pathways involved in mediating responses to mechanical loading can be used to select improved protocols for distraction osteogenesis, a surgical technique used to treat bony deformities via limb lengthening. Finally, in their paper, Betts and Müller review the different experimental studies, models, and theories that explain how mechanical stimuli are sensed and incorporated during bone regeneration as part of the healing process after bone injury.

## Conclusion

Together, the 11 papers published in this Research Topic provide a good illustration of how mechanical loading underpins many facets of bone biology, including the pathogenesis and treatment of osteoporosis and other clinical disorders associated with skeletal fragility.

## Conflict of Interest Statement

The author declares that the research was conducted in the absence of any commercial or financial relationships that could be construed as a potential conflict of interest.

